# Experimental validation and computational modeling of anti-influenza effects of quercetin-3-O-α-L-rhamnopyranoside from indigenous south African medicinal plant *Rapanea melanophloeos*

**DOI:** 10.1186/s12906-019-2774-3

**Published:** 2019-12-02

**Authors:** Parvaneh Mehrbod, Samad Nejad Ebrahimi, Fatemeh Fotouhi, Fatemeh Eskandari, Jacobus N. Eloff, Lyndy J. McGaw, Folorunso O. Fasina

**Affiliations:** 1 Influenza and Respiratory Viruses Department, Pasteur Institute of Iran, Tehran, Iran; 2 Department of Veterinary Tropical Diseases, University of Pretoria, Pretoria, South Africa; 3 Department of Phytochemistry, Medicinal Plants and Drugs Research Institute, Shahid Beheshti University, Tehran, Iran; 4 Phytomedicine Programme, Department of Paraclinical Sciences, University of Pretoria, Pretoria, South Africa; 5 ECTAD, Food and Agriculture Organization of the United Nations (FAO), Dar es Salaam, Tanzania

**Keywords:** Influenza a virus, Quercetin-3-O-α-L-rhamnopyranoside, Cytokine, Apoptosis, Molecular docking

## Abstract

**Background:**

Influenza A virus (IAV) is still a major health threat. The clinical manifestations of this infection are related to immune dysregulation, which causes morbidity and mortality. The usage of traditional medication with immunomodulatory properties against influenza infection has been increased recently. Our previous study showed antiviral activity of quercetin-3-O-α-L-rhamnopyranoside (Q3R) isolated from *Rapanea melanophloeos* (RM) (L.) Mez (family Myrsinaceae) against H1N1 (A/PR/8/34) infection. This study aimed to confirm the wider range of immunomodulatory effect of Q3R on selective pro- and anti-inflammatory cytokines against IAV in vitro, to evaluate the effect of Q3R on apoptosis pathway in combination with H1N1, also to assess the physical interaction of Q3R with virus glycoproteins and RhoA protein using computational docking.

**Methods:**

MDCK cells were exposed to Q3R and 100CCID_50_/100 μl of H1N1 in combined treatments (co-, pre- and post-penetration treatments). The treatments were tested for the cytokines evaluation at RNA and protein levels by qPCR and ELISA, respectively. In another set of treatment, apoptosis was examined by detecting RhoA GTPase protein and caspase-3 activity. Molecular docking was used as a tool for evaluation of the potential anti-influenza activity of Q3R.

**Results:**

The expressions of cytokines in both genome and protein levels were significantly affected by Q3R treatment. It was shown that Q3R was much more effective against influenza when it was applied in co-penetration treatment. Q3R in combination with H1N1 increased caspase-3 activity while decreasing RhoA activation. The molecular docking results showed strong binding ability of Q3R with M2 transmembrane, Neuraminidase of 2009 pandemic H1N1, N1 and H1 of PR/8/1934 and Human RhoA proteins, with docking energy of − 10.81, − 10.47, − 9.52, − 9.24 and − 8.78 Kcal/mol, respectively.

**Conclusions:**

Quercetin-3-O-α-L-rhamnopyranoside from RM was significantly effective against influenza infection by immunomodulatory properties, affecting the apoptosis pathway and binding ability to viral receptors M2 transmembrane and Neuraminidase of 2009 pandemic H1N1 and human RhoA cellular protein. Further research will focus on detecting the detailed specific mechanism of Q3R in virus-host interactions.

## Background

Influenza A virus (IAV) is a respiratory tract pathogen that causes a high number of deaths and hospitalizations, including approximately 49,000 deaths and up to 35,600,000 morbidity cases annually in the USA alone [1, 2]. IAV recruits host cell machinery to support their replication and transportation inside the cell [3, 4]. In this regard, targeting cellular proteins in virus-host interaction pathways could be effective against influenza infection. The benefit of this approach is to reduce virus drug resistance. However, this strategy requires more understanding of the intracellular pathways that influenza A virus uses to replicate [3].

Several studies have suggested an association between inflammation and severe cases of IAV infection [5–7]. The host inflammatory response following influenza virus infection presents the host cells and the immune system with somewhat overwhelming effects. Host inflammatory response is a key factor in controlling virus proliferation but is also associated with lung damage, morbidity and death in the case of overwhelming inflammation [8]. Uncontrolled and exacerbated response to the virus may be associated with intense lung injury and death [9, 10].

In view of the foregoing, a growing number of studies have suggested that immunomodulatory strategies may improve disease outcome without affecting the ability of the host to deal with infection [11, 12]. Herbal medications have equally attracted tremendous attention [13, 14], as complementary therapies and preventive medicine [15–18].

Rho GTPase molecules, which can be modified post-translationally, control a wide variety of signal transduction pathways. They can affect cell polarity, microtubule dynamics, membrane transport and transcription factor activities [19]. They are well-known proteins to regulate the intracellular signal transducers [20–22]. RhoA is one of the best-studied members of this family which is widely documented as a key regulator of cytoskeletal dynamics reorganization on membrane trafficking [23, 24]. Increasing evidence suggests a link between Rho proteins and apoptosis [25–27]. Several studies have also focused on the therapeutic effects of flavonoid compounds through Rho proteins expression and apoptosis pathway [28–30]. Different types of flavonoids have been identified as antiviral agents [31–34]. The reports of antiviral activity of flavonoids and derivatives against several viral infections through different mechanisms are increasing such as Adenovirus, Herpes Simplex Virus, Japanese Encephalitis Virus, Respiratory Syncytial Virus, HIV-1, Dengue Virus, Hepatitis C Virus and Zika Virus [35–41]. The potent antiviral effect of flavonoids against influenza virus infection [42–44] and immunomodulatory effects of flavonoids in different viral infections [45–47] have been reported as well.

Quercetin from the flavonoid group of plant compounds has been studied in small clinical trials [48]. There are limited studies on immunomodulatory effects of quercetin on influenza infection. One such study indicated the inhibitory activity of quercetin on influenza infection in the early stage of entry [49]. Q3R from *Houttuynia cordata* demonstrated strong anti-influenza A/WS/33 virus activity, reducing the formation of visible CPE, and inhibited virus replication in the initial stage of virus infection [50].

The biological activity of flavonoids depends on the configuration, the total number of hydroxyl groups, and substitution of functional groups about their nuclear structure [34].

Quercetin belongs to the class called flavonols that cannot be produced in the human body but only in plant material and products [51]. It is one of the important flavonoid compounds isolated from more than twenty plant material from USA, Europe, and eastern countries which is known for its different properties especially anti-inflammatory activities [52, 53]. We also reported quercetin isolation from *Rapanea melanophloeos* (Myrsinaceae family) an indigenous South African plant for the first time [54].

In continuation of our previous study [54], this research was designed to confirm and reveal the additional immunomodulatory activity of Q3R and its effect on the apoptosis pathway, in controlling influenza infection. Computational molecular docking was also performed to screen the potential binding ability of Q3R with neuraminidase/hemagglutinin glycoproteins and M2 transmembrane from H1N1, and Human RhoA.

## Materials and methods

### Immunomodulatory evaluation

The quercetin-3-O-α-L-rhamnopyranoside was isolated from *Rapanea melanophloeos* [55]. The antiviral activity of Q3R against influenza infection was evaluated in our earlier study [54]. The non-cytotoxic concentration (NCTC) of Q3R (150 μg/ml) was exposed to the cell in combination with 100CCID_50_/100 μl of H1N1. Its immunomodulatory capacity was confirmed by testing cell-free supernatants treated for 48 h pointing at TNF-α and IL-27 previously [54]. In this study, IL-6 and CCL-2 as pro-inflammatory cytokines and IFN-β as anti-inflammatory cytokines were measured additionally at RNA and protein levels by qPCR and ELISA, respectively to add more values to Q3R immunomodulatory properties profile.

The molecular assay was conducted as stated before [54]. The primers specifications are shown in Table 1. The primers used for housekeeping genes were mentioned in our previous study [54].

**Table 1:** The specification of the primers for amplification of the targeted genes

Gene name	Primer sequence (5′ to 3′)	Accession number	Position	Size (bp)	Tm (°C)	Optimized annealing temperature (Ta) (°C)
IL-6-F	GTTCGGATAATGTAGCCT	NM_001003301.1	633–650	135	40.6	53.9
IL-6-R	TCACAGAGAACAACATAACT		751–768		40.5	
CCL-2-F	GTGATCTTCAAGACCGTCCTAA	NM_001003297.1	191–212	130	47.9	59.5
CCL-2-R	TTCAGAGTGAGTATTCATGGCTT		299–321		46.6	
IFN-β-F	AAACTTCACCTGGGACAA	NM_001135787.1	390–407	118	40.6	55.9
IFN-β-R	TTTCTGCTTGGACTATTGT				39.5	

The IL-6 cytokine protein was quantified by quantitative sandwich Picokine ELISA kits (Boster Biological Technology, CA, USA) according to the manufacturer’s instruction as stated previously [54]. The IFN-β and CCL2 were evaluated by sandwich geneILNB1 kit (EIAab Science Co, China) and sandwich Ready-SET-Go kit (Invitrogen, USA) according to the manufacturers’ instructions, respectively. The optical densities were measured at 450 nm wavelength using microplate reader (Anthos 2020, version 2.0.5). The concentrations were calculated according to the corresponding reaction standard formula. All the data were statistically analyzed by SPSS version 22. Analysis of variance (ANOVA), Post hoc Tukey test was used to determine the significance of difference among the treatments (*P* < 0.05).

### Sample preparation for protein evaluation

MDCK cells were cultured in T-75 flasks (Orange Scientific, Belgium). The cells were simultaneously treated with Q3R (150 μM) and Y-27632 (10 μM) (RhoA inhibitor) in the presence or absence of H1N1 (100 TCID_50_/0.1 ml) for 48 h. Untreated cells and virus-inoculated cells were considered as negative and positive controls, respectively. Cells were scraped and collected in ice cold PBS and centrifuged to form pellet. The cell pellet was re-suspended and homogenized in RIPA buffer mixed with protease inhibitor cocktail. The homogenate was centrifuged (15,000 g 4 °C 15 min). The pellet was discarded and supernatant was stored at − 80 °C for further evaluation. The protein concentration in each sample was quantified using Bradford Assay by Microplate reader (BioTek EL 800, US) [56].

#### RhoA protein immunoblotting

The protein samples (50 μg) were fractioned using 15% sodium dodecyl sulfate polyacrylamide gel electrophoresis (SDS-PAGE) and transferred to nitrocellulose membrane (Bio-Rad) by vertical semi-dry electroblotting. Following 1 h blocking of the membranes using 1% BSA as blocking buffer and washing steps with Tris-buffered saline (TBS)-Tween, the membranes were incubated overnight with primary antibodies (Rabbit primary polyclonal anti-RhoA, Merck, Germany, 1:1000; and Rabbit primary monoclonal anti-β-actin, Cell Signaling Technology, USA, 1:1000) at 4 °C. Followed by washing, the membranes were exposed to AP-conjugated secondary antibody (Goat polyclonal anti-Rabbit IgG, Abcam, UK, 1:1000) for 2 h at room temperature. The enzymatic reaction was visualized using a BCIP-NBT substrate (Merck, Germany). The β-actin was detected as housekeeping protein for loading control. The blots were scanned and the protein bands intensities were quantified using Image J 1.46r software. For each sample, signal intensity was normalized to the negative control and internal control.

#### Caspase-3 cellular activity assay

The cells were cultured in T-75 flasks (Orange Scientific, Belgium) and treated as mentioned above. Untreated cells were considered as negative control. The caspase-3 included in the kit was used as a positive control. Following cell lysis, the proteins were extracted and quantified using Bradford Assay. The conversion factor of the reader was determined before beginning the assay. The caspase-3 Cellular Activity Assay Kit (Merck, Germany) was used for this test. The substrate standard [50 μM p-nitroaniline (pNA) in the assay buffer] (100 μl) was added to 2 wells of the plate. The average A405 using 100 μl assay buffer as a blank was determined. This calculation was based on the concentration of p-nitroaniline in the calibration standard (50 μM).

For caspase-3 cellular activity evaluation in the samples, the protein extracts were added to the 96-well plate (10 μl) which already had assay buffer (40 μl). A well with only assay buffer was used as blank. The plate was incubated at room temperature for 10 min to allow inhibitor/enzyme interaction. The reaction was started by adding 50 μl caspase-3 Substrate I, Colorimetric. The absorbance was measured at 405 nm. Data were recorded every 10 min for 30 min after adding the substrate.

### Statistical analysis

Data presented as means ± SD were analyzed using SPSS 18.0. One-way analysis of variance (ANOVA) post-hoc LSD test was used to analyze the effect of treatments on RhoA protein expression. All charts were plotted using GraphPad Prism 5.0. For all tests, *P* ≤ 0.05 was considered significant.

### Molecular docking

The 2D structures of the compound were created by ChemDraw Professional 15.0 and 3D structures were generated by Chem3D suite. The SDF format of structures was saved and used for docking study. The energy minimization of the 3D structure was performed by Lig Prep module implemented in Schrodinger 2015–2, using Maestro 10.2 platform. The crystal structures of influenza neuraminidase of A/Brevig Mission/1/1918 H1N1 strain (3BEQ), PR/8/1934 human strain H1 hemagglutinin (1RU7), avian H5 haemagglutinin (1JSN), 2009 pandemic H1N1 neuraminidase (3TI6), M2 transmembrane (2KQT), N1 neuraminidase (2HTY) and Human RhoA (1A2B) were selected from the previous publications [57, 58] and downloaded from protein data bank (www.rcsb.org). The protein preparation for docking was performed using Protein Preparation Wizard on Maestro 10.2 Schrodinger suite. The protein preparation included deleting of water molecules, assigning formal charge and bond order of protein, adding all missing hydrogen atoms, loops and side chains to crystal structures. Then the hydrogen network was refined and hydrogen minimization was carried out by OPLS3 force field parameter. The receptor grid box was generated with a box size of 25 Å × 25 Å × 25 Å using the Grid generation application. The Q3R and native ligands Oseltamivir, Rimantadine, Arbidol, Guanosine 5′-[β,γ-imido]triphosphate (GMP-PNP, Gpp(NH)p) were docked to grid files of proteins by Glide extra precision (XP) mode.

## Results

### Relative expression analysis

**Fig. 1:**
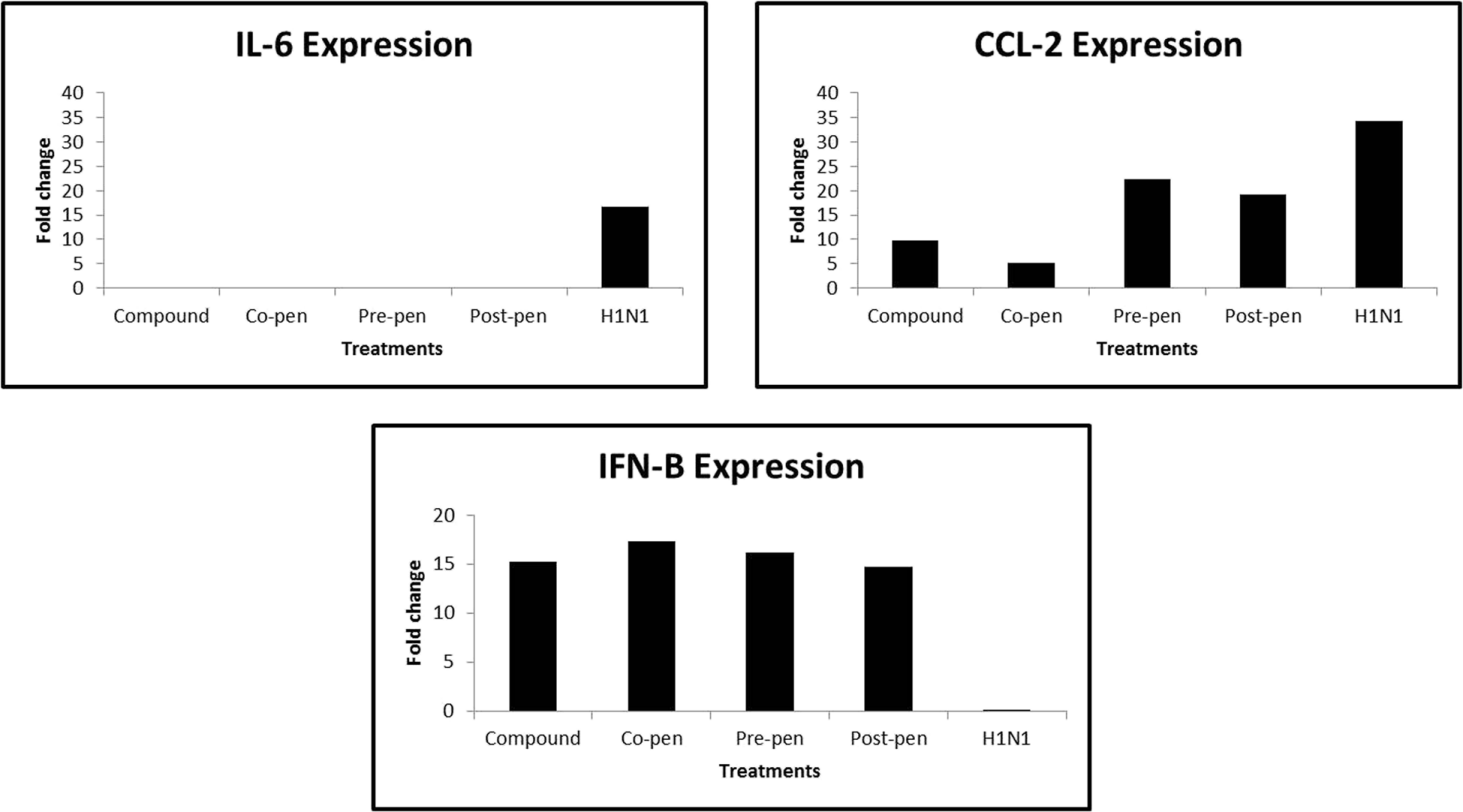
Relative expression analysis (ΔΔCq) of the cytokines compared to the positive control

The relative expression analysis of the cytokines genes was calculated as fold change compared to the positive control. Data are shown in Fig. 1. As observed in the Figure, H1N1 inoculation increased IL-6 and CCL-2 expressions 16.62 and 34.18 fold, respectively, but in combination treatments, they all showed decrements, especially in the co-penetration procedure the compound decreased IL-6 and CCL-2 expressions highly significantly 0.0004 and 5.2221 fold, respectively.

With regards to IFN-β, H1N1 inoculation decreased this cytokine 0.0004 fold while it increased 16.11 fold on average for all combined treatments with no significant difference as compared with each other.

### Cytokines analysis with ELISA

The levels of cytokines proteins in supernatants of MDCK cell culture at 48 h after exposure were detected. The percentage of changes of all cytokines is shown in Table 2. As shown in the Table 2, all combination treatments were effective in modulating the cytokine protein levels, especially in the co-penetration procedure.

**Table 2:** Cytokines protein percentages of changes in MDCK culture supernatants at 48 h treatment

Treatment	IL-6% change to H1N1	CCL-2% change to H1N1	IFN-β % change to H1N1
Co-pen	−76.659	− 52.488	− 10.511
Pre-pen	− 49.937	−22.761	4.955
Post-pen	−45.553	−10.607	−5.706

Percentages of cytokines changes compared to H1N1, as determined by ELISA, are expressed as pg/ml (*N* = 2) for 48 h incubation time

### RhoA immunoblotting results

**Fig. 2:**
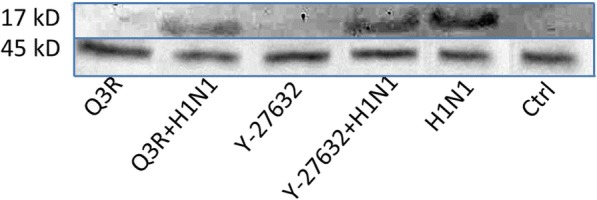
Modulation of the RhoA protein expression. MDCK cells were treated with Q3R and Y-27632 in the presence or absence of H1N1 for 48 h. The blot is typical of two independent experiments with similar results. Each blot was scanned and imaged separately

The expression level of RhoA protein was affected by different treatments (Fig. 2). As shown in the Western Blot image, Q3R treatment depleted this protein expression. The increase of RhoA in the H1N1-inoculated sample was evident. The combination treatment of Q3R and H1N1 showed increment in the expression of RhoA but not so much as H1N1 sample. Y-27632 is a selective inhibitor of Rho-associated protein kinases also decreased RhoA protein expression.

**Fig. 3:**
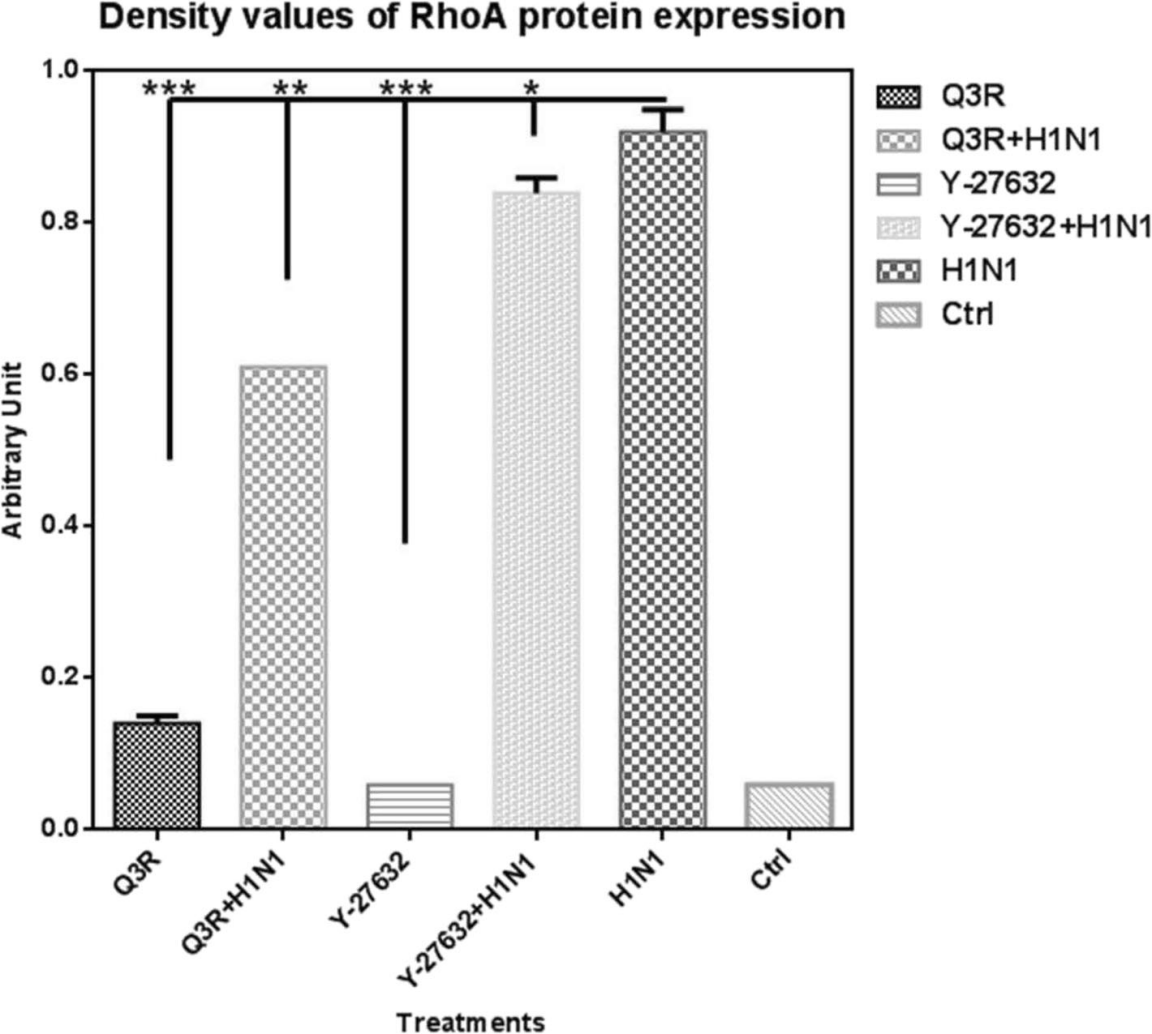
Density values of RhoA protein expression. Values show the density of the RhoA protein bands normalized to the related housekeeping protein (β-actin) quantified by ImageJ. (***P ≤ 0.001; **P ≤ 0.01; *P ≤ 0.05)

Band densitometry and statistical analysis (Fig. 3) verified that in comparison with the H1N1-inoculated sample, Q3R treatment depleted this protein expression highly significantly (*P* < 0.001). The combination treatment of Q3R and H1N1 decreased the expression of RhoA significantly (*P* < 0.01). Β-actin as housekeeping protein remained unchanged after the treatments.

### Caspase-3 cellular activity assay results

**Fig. 4:**
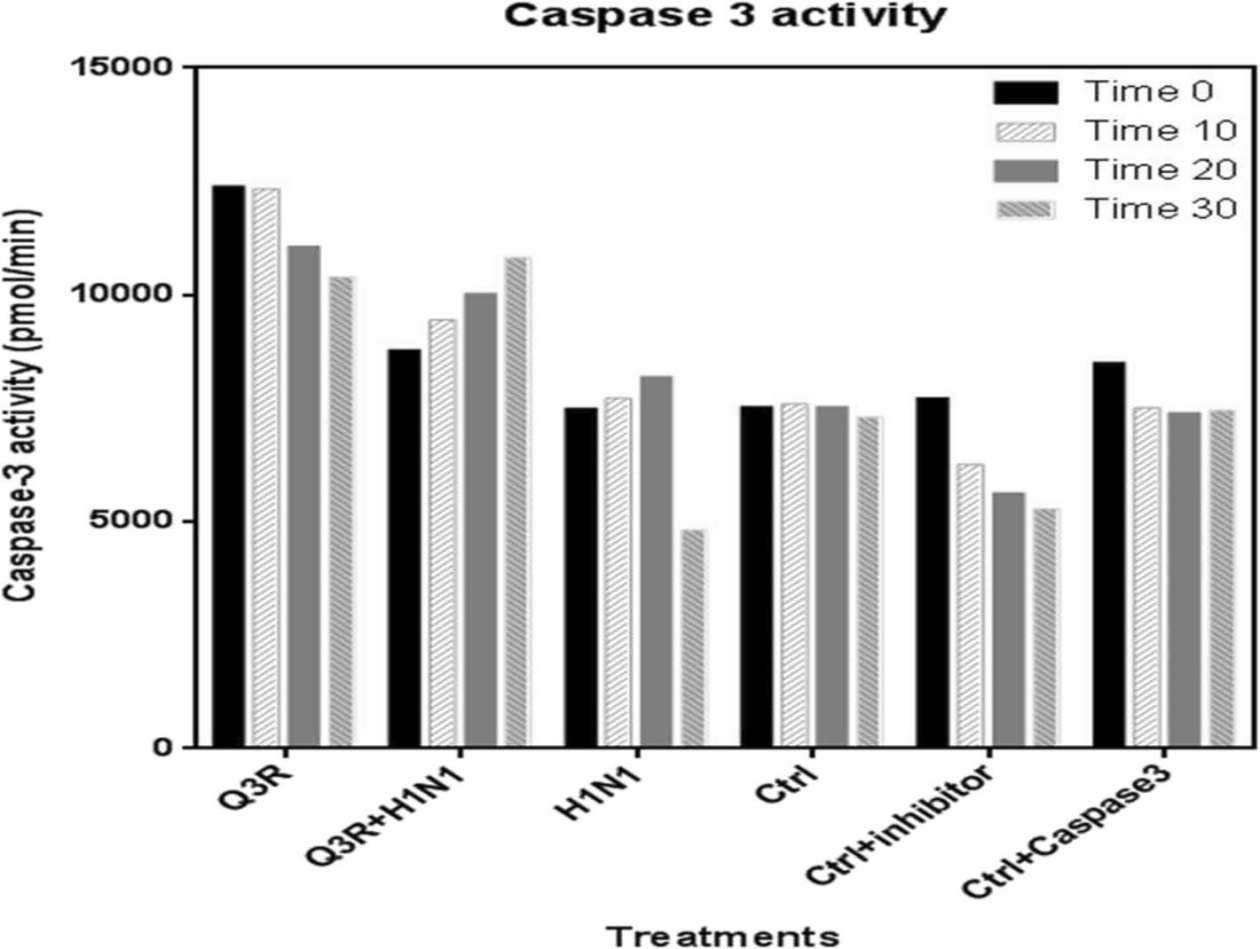
Caspase-3 activity evaluation

In this assay, the effect of Q3R and H1N1 was evaluated on the caspase-3 activity. As shown in Fig. 4, Q3R treatment increased caspase-3 activity with a slight decrement by increasing time but showed the highest values compared to H1N1. However, in H1N1-inoculated sample, caspase-3 activity showed decline, which is indicative of RhoA activity increment by the virus. The combination treatment of Q3R with H1N1 showed intermediate values of caspase-3 activity in all incubation times with a slight increment by increasing time.

### Molecular docking results

**Fig. 5:**
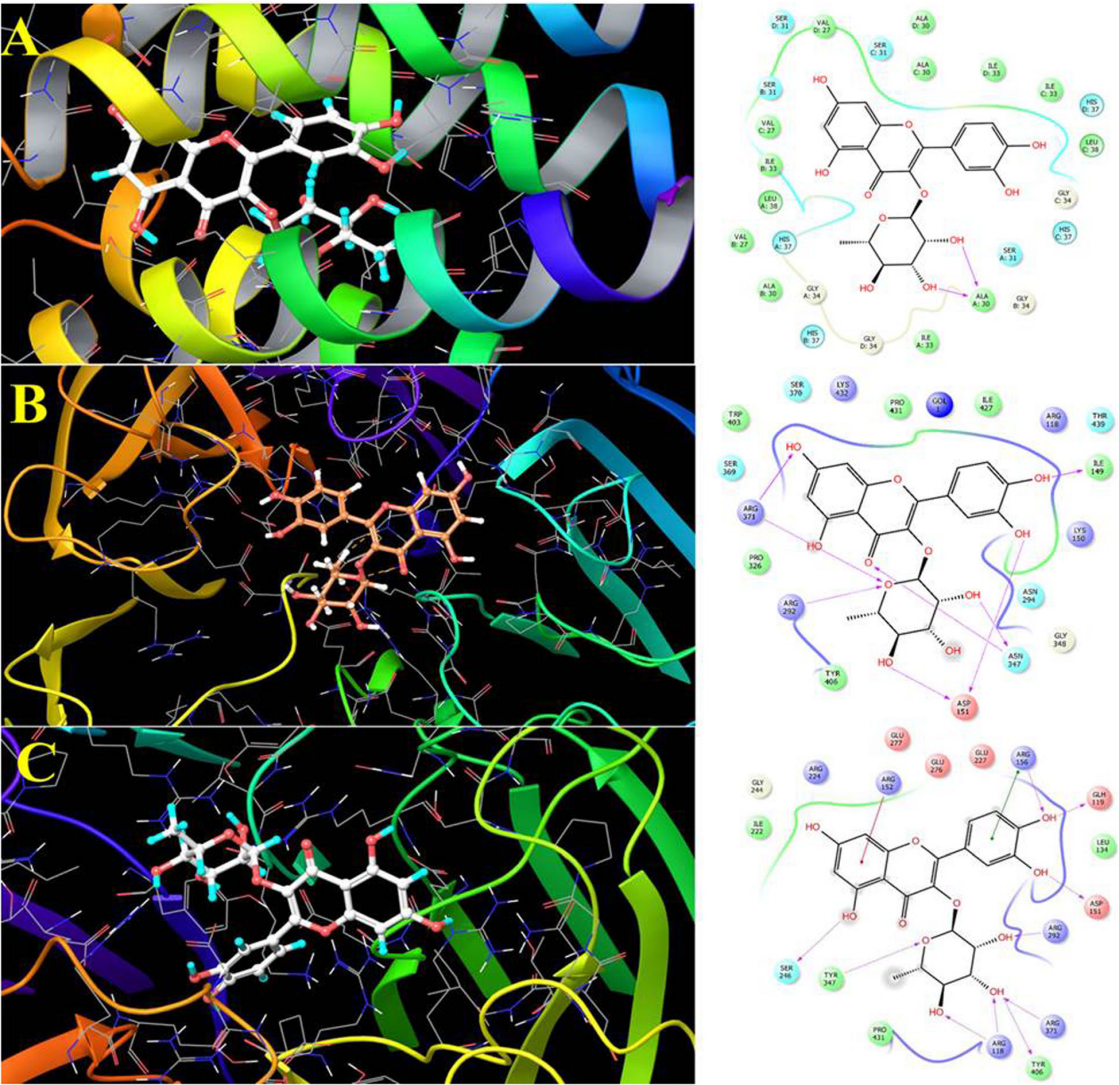
The 2D interaction and 3D docking model of Q3R with **a**) M2 transmembrane (2KQT) and **b**) Neuraminidase 2009 pandemic H1N1 (3TI6) receptors from influenza virus and **c**) human Ras homolog family member A (RhoA). The docking was performed using Glide application using Schrödinger drug discovery suite

The interaction between Q3R and influenza hemagglutinin/neuraminidase/ M2 and RhoA proteins was carried out by molecular docking study. For this purpose, five crystal structures were selected based on the previously published data described in the experimental section. The results of interactions and docking scores were reported in Table 3. The Q3R showed excellent docking score with M2 transmembrane (2KQT), Neuraminidase of 2009 pandemic H1N1 (3TI6), N1 neuraminidase (2HTY) and PR/8/1934 Human strain H1 hemagglutinin (1RU7) by the values of − 10.81, − 10.47, − 9.52 and − 9.24 kcal/mol, respectively. Also the interaction of Q3R and RhoA (1A2B) showed high affinity with docking score of − 8.78 Kcal/mol comparing to the GMP-PNP, Gpp(NH)p with value of − 5.55 Kcal/mol, the strong hydrogen bonding between Q3R and GLH119, ASP151, ARG292, TYR406 and SER249 were involved in this high affinity (Fig. 5c). The Q3R showed a much better docking score with studied targets comparing to the antiviral drugs oseltamivir, rimantadine, arbidol and GMP-PNP, Gpp(NH)p as positive controls (Table 3).

**Table 3:** The docking energy of Q3R with the studied receptors

Protein (PDB ID)	Docking energy (Kcal/mol)				
	Q3R	Oseltamivir	Rimantadine	Arbidol	GMP-PNP, Gpp(NH)p
Neuraminidase of A/Brevig Mission/1/1918 (3BEQ)	−6.58	− 7.40			
PR/8/1934 Human H1 (1RU7)	−9.24			−4.51	
Hemagglutinin of Avian H5 (1JSN)	−6.87	−8.08			
Neuraminidase of 2009 pandemic H1N1 (3TI6)	−10.47	−7.06			
M2 transmembrane (2KQT)	−10.81		−5.51		
N1 neuraminidase (2HTY)	−9.52	−4.68			
Human RHOA (1A2B)	−8.78				−5.55

## Discussion

The modification and manupulation of cytokines production is a critical step in influenza pathogenesis, which can recruit a variety of innate immune cells [59]. In this study, in completing our previous study [54, 60], interleukin-6 (IL-6) and chemokine C-C motif ligand 2 (CCL-2) from the category of pro-inflammatory cytokines and interferon-β (IFN-β) from the category of anti-inflammatory cytokines were tested at the genome and protein levels.

The cytokine production was affected by quercetin during influenza course. Q3R was able to significantly decrease the IL-6 production to 0.0004, 0.0202 and 0.0733 fold in co-, pre- and post-penetration treatments, respectively at the genome level (Fig. 1). This decrement was observed in protein level as well. However, the co-penetration treatment showed more elimination to − 76.66% (Table 2). This cytokine is highly correlated with high body temperature during influenza illnesses and infection [61, 62], and strong up-regulation of this cytokine can predict the severity of the infection [63]. Consequently, Q3R has a high potential to decrease the fever and severity of influenza illnesses by regulating the excessive innate inflammatory reaction.

Chemokine (C-C motif) ligand 2 (CCL-2) was one of the affected chemokines evaluated in this study. This chemokine recruits memory T cells, exudate macrophages (exMACs) and monocytes to the site of the infection [60, 63, 64]. Attraction of these CCR2^+^ inflammatory cells to the site would lead to naive T cell proliferation, NO synthesis 2 (NOS2) and TNF-α production [63]; however, the extreme recruitment of these cells causes extra cytokine production and apoptosis induction by tumor necrosis factor-related apoptosis inducing legend (TRAIL) activation [65]. In the children with deadly acute encephalopathy associated with IAV, high concentrations of CCL-2 and CSF have been reported [66]. Decrement in CCL-2 and mononuclear cells in the infection site can effectively control the virus infection by improving the condition without affecting the CD8^+^ T cell expansion [67]. In the current study, Q3R showed the ability to significantly decrease the CCL-2 protein production and gene expression especially in the co-penetration treatment to − 52.488% (Table 2) and 5.2221 fold (Fig. 1), respectively compared to H1N1 inoculation which can decrease the mortality associated with the highly pathogenic influenza viruses.

IFN-β is a type I interferon and inhibitor of inflammation [68]. It can shift the cytokine networks in favor of anti-inflammatory effects [69]. In the study of protein level, H1N1 showed a mild increase in IFN-β protein compared to the negative control, however, no significant difference was observed at the protein level between H1N1 inoculation and combined treatments. The gene expression of IFN-β decreased in H1N1 sample by 0.0004 fold but in all combined treatments it showed increments at the same level (Fig. 1). This can be referred to excessive intracellular viral NS1 protein that prevents the induction of beta interferon [70]. In addition, referred to our previous study which showed elevated levels in TNF-α production in H1N1 sample and its significant decrement when subjected to Q3R [54], it is reported that increase of TNF-α can inhibit the production of IFN α/β [54]. This outcome might be the reason for this effect of Q3R which shows that Q3R does not act through IFNs.

Thus, the compound Q3R could interrupt the effect of the influenza virus on the cytokines which could decrease pro-inflammatory and increase anti-inflammatory cytokines.

It has been studied that active RhoA prevents endothelial apoptosis. Therefore, inhibition of Rho in endothelial cells not only reduced the expression of anti-apoptotic Bcl-2 and Mcl-1 and increased proapoptotic Bid protein levels, but also, activated caspase-9- and − 3-dependent programmed cell death (apoptosis) [25]. A recent study highlighted that RhoA might be important for the proliferation and apoptosis in lung cancer cells. Alterations in caspase-3 may be the underlying molecular mechanisms associated with the effect of RhoA on cell proliferation and apoptosis [71].

Quercetin is a flavanol compound occurring naturally in plant materials. It increased the expressions of RhoA and Rho-associated, coiled-coil containing protein kinase-1 (ROCK1), but inhibited the expression of NF-κB p65 in SAS Human oral cancer cells. This role of quercetin was mentioned to be associated with the down-regulation of PKC and RhoA by blocking MAPK and PI3K/AKT signaling pathways and NF-κB [29]. Rho GTPase-mediated mechanisms of isoquercitrin were tested in a study. It was shown that RhoA underwent translocation to the cytoplasm upon treatment with isoquercitrin. Thus, it affected RhoA localization preventing the translocation to the plasma membrane [28]. Flavonoids have also shown protection against apoptosis on neurons involving c-Jun N-terminal kinase (JNK), c-Jun and caspase-3 [30].

Different quercetin derivatives may play different roles in signaling pathways depending on the type of cell or disease. The effect of quercetin-3-O-(2″-galloyl)-α-l-rhamnopyranoside (QGR) was investigated on TRAIL-induced apoptosis in human keratinocytes. Treatment with QGR prevented TRAIL-induced apoptosis-related protein activation, but quercetin had an additive effect on TRAIL-induced apoptosis-related protein activation and cell death [72]. In a recent study, quercetin-3-O-α-L-rhamnopyranoside (Q3R) decreased the oxidative stress in human umbilical vein endothelial cells (HUVECs) by promoting the nuclear transfer of nuclear factor erythroid 2-related factor 2 (Nrf2) and heme oxygenase-1 by activating autophagy. This study supported the use of quercetin-3-O-α-L-rhamnopyranoside (Q3R) as a health supplement to alleviate oxidative stress [73].

In regards to apoptosis pathway and RhoA and apoptosis interaction [25], it was found in this study that in combination treatment of Q3R with H1N1, RhoA expression decreased and caspase-3 activity increased compared to the H1N1 sample. Caspase-3, a member of the interleukin-1β converting enzyme (ICE) family of cysteine proteases, is one of the principal caspases found in the apoptotic cells. The highest values of caspase-3 activity in Q3R sample compared to H1N1 correlates with the RhoA decrement in Q3R treatment. And intermediate value of caspase-3 activity in combination treatment of Q3R with H1N1 was indicative of regulatory role of Q3R on RhoA activity against viral molecular pathway.

The molecular docking results showed strong binding ability of Q3R with M2 transmembrane, Neuraminidase of 2009 pandemic H1N1, N1 neuraminidase, PR/8/1934 Human strain H1 and Human RhoA, with docking energy of − 10.81, − 10.47, − 9.52, − 9.24 and − 8.78 Kcal/mol, respectively. Regarding the docking study, there are three essential types of interactions with the enzyme active site including hydrogen bond, Pi-Pi staking, and hydrophobic interactions. The Q3R showed strong interactions with M2 transmembrane (2KQT) residues, namely with ALA A30, ILE B33, VAL D27, VAL C27, ALA D30, phenolic and sugar moieties (Fig. 5a). The Q3R showed several hydrogen-bonding interactions with 3TI6 receptors, namely with ASP151, ASN347, and ILE149, together with Pi-Pi staking with ILE149, ILE427, PRO431, TRP403 and TYR406 from phenolic and sugar moieties. This implies that the presence of both parts is essential for high affinity with ligand and receptor.

## Conclusions

In conclusion, while our previous studies showed the safety effects of Q3R over amantadine and oseltamivir, and also supporting data from the current study, in vitro evaluation of the consequences of Q3R revealed this natural compound has the potential to alleviate influenza infection by modulating the inflammatory response, affecting the apoptosis pathway and efficiently improve the outcome of the influenza disease.

Further details on the docking results showed that the structural features of Q3R might be helpful for further drug design and development. We concluded that Q3R can inhibit the virus affecting the virus penetration/adsorption directly. Correspondingly, it showed the ability to indirectly dominate the severity of the disease by changing the cytokines pattern and affecting the apoptosis pathway. Thus, the concurrent application of quercetin-3-O-α-L-rhamnopyranoside with influenza A virus infection is highly effective in influenza infection modulation. Therefore, understanding and targeting the cellular proteins and intracellular pathways required for influenza replication are valuable and beneficial to prevent or treat this infection more efficiently. Further in vivo evaluation can also assist in understanding the benefits of quercetin against influenza disease in a perceptible way.

## Data Availability

The datasets used and/or analyzed during the current study are available from the corresponding author on reasonable request.

## Abbreviations

ANOVA: Analysis of variance
CCID_50_: Cell culture infectious dose 50
CCL-2: Chemokine C-C motif ligand 2
ELISA: Enzyme-linked immunosorbent assay
exMACs: Exudate macrophages
HA: Hemagglutination
HUVECs: Human umbilical vein endothelial cells
IAV: Influenza A virus
ICE: Interleukin-1β converting enzyme
MDCK: Madin Darby Canine Kidney
NA: Neuraminidase
NCTC: Noncytotoxic concentration
NOS2: NO synthesis 2
Nrf2: Nuclear factor erythroid 2-related factor 2
OD: Optical Density
PBS: Phosphate-buffered saline
Q3R: Quercetin-3-O-α-L-rhamnopyranoside
QPCR: Quantitative PCR
RM: Rapanea melanophloeos
TRAIL: Tumor necrosis factor-related apoptosis-inducing legend
Trypsin-TPCK: Tosylamide Phenylethyl Chloromethyl Ketone-treated Trypsin
